# Single-Frequency GNSS Integer Ambiguity Solving Based on Adaptive Genetic Particle Swarm Optimization Algorithm

**DOI:** 10.3390/s23239353

**Published:** 2023-11-23

**Authors:** Ying-Qing Guo, Yan Zhang, Zhao-Dong Xu, Yu Fang, Zhi-Wei Zhang

**Affiliations:** 1 College of Mechanical and Electronic Engineering, Nanjing Forestry University, Nanjing 210037, China; zyagwx@njfu.edu.cn (Y.Z.); zhiwei_zhang@njfu.edu.cn (Z.-W.Z.); 2 China-Pakistan Belt and Road Joint Laboratory on Smart Disaster Prevention of Major Infrastructures, Southeast University, Nanjing 210096, China; xuzhdgyq@seu.edu.cn (Z.-D.X.);

**Keywords:** carrier phase measurement, global navigation satellite system (GNSS), integer ambiguity, adaptive genetic particle swarm optimization (AGPSO)

## Abstract

Carrier phase measurements currently play a crucial role in achieving rapid and highly accurate positioning of global navigation satellite systems (GNSS). Resolving the integer ambiguity correctly is one of the key steps in this process. To address the inefficiency and slow search problem during ambiguity solving, we propose a single-frequency GNSS integer ambiguity solving based on an adaptive genetic particle swarm optimization (AGPSO) algorithm. Initially, we solve for the floating-point solution and its corresponding covariance matrix using the carrier-phase double difference equation. Subsequently, we decorrelate it using the inverse integer Cholesky algorithm. Furthermore, we introduce an improved fitness function to enhance convergence and search performance. Finally, we combine a particle swarm optimization algorithm with adaptive weights to conduct an integer ambiguity search, where each generation selectively undergoes half-random crossover and mutation operations to facilitate escaping local optima. Comparative studies against traditional algorithms and other intelligent algorithms demonstrate that the AGPSO algorithm exhibits faster convergence rates, improved stability in integer ambiguity search results, and in practical experiments the baseline accuracy of the solution is within 0.02 m, which has some application value in the practical situation of short baselines.

## 1. Introduction

In the Global Navigation Satellite System (GNSS), achieving highly accurate positioning results heavily relies on utilizing carrier phase observations to calculate the receiver-to-satellite distance. However, during carrier phase observation, the receiver can only measure the non-integer portion of the carrier phase, and each measurement introduces an unknown constant referred to as the integer ambiguity [1]. Consequently, resolving this ambiguity is crucial for attaining fast and precise localization. Ambiguity resolution involves converting floating-point resolution into integer values; when correctly determining the integer ambiguity of the carrier phase, localization accuracy at the centimeter or even millimeter level can be achieved. Conversely, an incorrect determination of this ambiguity will lead to jumps in localization results due to deviations in ambiguity. For GNSS-RTK positioning, the accuracy and stability of fixing this integer ambiguity determine both positioning accuracy and reliability. Therefore, resolving the integer ambiguity has become a prominent research focus within GNSS positioning and navigation.

Over the decades, a variety of algorithms have emerged for solving integer ambiguity problems, including the Least Squares Ambiguity Search Method (LSAST) [2], Fast Ambiguity Solving Algorithm (FARA) [3], Least Squares Ambiguity Decorrelation Leveling Method (LAMBDA) [4,5], Fast Ambiguity Search Filter (FASF) [6], Triple-Frequency Carrier Ambiguity Algorithms (TCAR) [7], and others. Among these methods, LAMBDA has gained widespread adoption and is considered the standard algorithm for ambiguity solving in most research centers. This algorithm consists of two main steps: firstly, employing the least squares method to obtain a floating solution for the ambiguity; secondly, transforming the ambiguity parameters and their covariance matrices from their original space to a new space using integer Gaussian transformation (also known as z-transformation), thereby achieving reduced correlation of the ambiguity. Finally, by searching for an optimal solution within this transformed space [4,5], fixation of the ambiguity is accomplished. Recently, significant advancements have been made by researchers in the field of the LAMBDA algorithm. Teunissen et al. [8] proposed a contraction method to reduce the search space. The basic idea is that once a new ambiguities vector group is obtained during the search process, its corresponding objective function value is calculated, and if the objective function value is less than $\chi^{2}$, the new $\chi^{2}$ value is equal to that objective function value. In this way, the search space is gradually reduced and an optimal ambiguities group is finally obtained. Chang et al. [9] proposed an enhanced method for integer least squares estimation: MLAMBDA, which enhances computational efficiency during the search phase by reducing the complexity of the LAMBDA method. PEI et al. [10] improved fixed integer ambiguity efficiency by establishing initial search space and optimally updating the upper and lower bounds of the LAMBDA algorithm. Wang et al. [11] introduced constraints based on known conditions to obtain accurate attitude information when float solutions and their variance–covariance matrices are insufficiently precise. The search ellipsoid region is expanded to compensate for errors caused by inaccurate floating solutions. Hu et al. [12] proposed an improved algorithm for determining the GPS/BDS dual-mode system’s integer ambiguity, utilizing Bootstrapping estimation to meet specific discriminative conditions, followed by an integer least-squares search after regularization limitation if these conditions are not met; this approach not only improves searching efficiency but also ensures accurate solution ambiguity. Ren et al. [13] proposed an enhanced LAMBDA method that improves the efficiency of solving ambiguity by modifying the previous approach of searching all ambiguity for each calendar element. By effectively combining search and direct normalization through reasonable conditions, this research enhances the determination of ambiguity after Z-transformation. These studies improve the efficiency of the LAMBDA algorithm by improving its search conditions and search space. The LAMBDA algorithm is theoretically rigorous, and although the fixation success rate is high, it is more time-consuming to compute and its search efficiency decreases as the dimensionality of the ambiguity increases. Teunissen et al. [14] investigated two simple alternatives for integer least squares estimation—rounding and bootstrapping —and calculated the probability of correct integer estimation. Although they are not optimal, they have the advantage that they do not require search at all in practical calculations, and the calculation is more efficient.

With the advancement of artificial intelligence, various algorithms such as the artificial fish swarm algorithm [15], simulated annealing algorithm [16], genetic algorithm [17], neural network algorithm [18,19,20], deep learning [21,22], and particle swarm optimization algorithm [23] have gained increasing attention and been applied by scholars to address the problem of integer ambiguity solving, significantly enhancing the efficiency of ambiguity search. Xu et al. [24] proposed an adaptive genetic algorithm-based search approach to resolve single-frequency GNSS carrier phase integer ambiguity, employing an adaptive genetic algorithm in the ambiguity searching process to enhance search efficiency. Li et al. [25] conducted an ambiguity search using an improved PSO (particle swarm optimization) algorithm. Tatiyaworanun et al. [26] proposed an ambiguity solving method based on a genetic algorithm with Grantham–Schmidt orthogonalization, effectively reducing both the dimensional search space and the number of searches. Liu et al. [27] suggested employing the Artificial Fish Swarm (AF) algorithm for efficient retrieval of integer ambiguity. Jazaeri et al. [28] investigated the effectiveness of ACO in addressing ambiguity and nearest grid point problems by rapidly resolving GNSS ambiguity through ant colony optimization. Zheng et al. [29] applied an adaptive differential evolutionary algorithm to find the optimal solution for integer ambiguity, subsequently constraining the region appropriately to maintain true integer ambiguity. Zheng et al. [30] proposed using genetic algorithms to solve weekly ambiguities by introducing two variance operators that ensure diversity among individuals. Xing et al. [31] proposed a combination of uniform design and genetic algorithm for searching the integer ambiguity. Wang et al. [32] solved the GPS short baseline integer ambiguity using an improved particle swarm optimization algorithm that encodes double difference ambiguity by real number encoding rounding and improves the success rate of ambiguity solving by adaptively calculating inertia weights and particle variances. Zhang et al. [33] proposed a DGPS integer ambiguity solving algorithm based on an improved particle swarm optimization algorithm that uses the inertia weight decreasing method of sin function to improve its particle swarm optimization weights. Li et al. [34] proposed a simulated annealing genetic algorithm-based approach for solving the integer ambiguity problem, which applies an improved genetic algorithm to search and solve the entire week’s uncertainty, ultimately obtaining optimal solutions for it. Liu et al. [35] introduced an improved ant colony algorithm for solving ambiguity throughout the entire week, which introduced a self-feedback factor based on traditional ant colony algorithms into their work; Liu et al. [27] presented a solution to this problem using artificial fish swarm algorithms with rounding improvements in their searches. Wang et al.’s study [36] used an enhanced artificial fish swarm technique with additional integer constraints to find fast fixation solutions. Ou et al. [37] modified the chicken flock optimization algorithm based on the ICSO algorithm to address the integer ambiguity and applied the enhanced chicken flock optimization algorithm for the integer ambiguity search. Shang et al. [38] proposed an improved PSO and ACO hybrid search algorithm for GNSS integer ambiguity, which utilizes the enhanced particle swarm optimization algorithm in the initial stage of coarse search to obtain a suboptimal solution, serving as an initialization for the pheromone distribution of the improved ant colony algorithm, ultimately achieving fine search for integer ambiguity. Deng et al. [39] introduced a week-long ambiguity-solving algorithm based on an adaptive differential evolution approach by incorporating an adaptive mutation operator, crossover operator, and population size into standard differential evolution to enhance the success rate of week-long ambiguity solving.

The application of the optimization algorithm to solving integer ambiguity enhances search efficiency compared to classical algorithms. However, these optimization algorithms possess inherent issues such as slow convergence speed, susceptibility to local optima, and other instabilities that result in unreliable ambiguity-solving outcomes. To address these concerns, this paper proposes a single-frequency GNSS integer ambiguity solving based on an adaptive genetic particle swarm optimization (AGPSO) algorithm. By leveraging the strengths of various optimization algorithms, this approach improves the stability of ambiguity solving and facilitates escape from local optima. Firstly, the carrier-phase double difference equation is employed to solve for floating-point solutions and their corresponding covariance matrix for integer ambiguity. Subsequently, an inverse integer Cholesky algorithm is utilized to reduce correlation while an improved fitness function is proposed to improve the convergence and search performance of the algorithm. Finally, the particle swarm optimization algorithm with adaptive weights is combined to perform the integer ambiguity search. Additionally, a selection mechanism is devised wherein the best-performing half of each generation is chosen based on fitness function values to proceed to the next generation. Furthermore, a random crossover operation is applied to the underperforming group by introducing a randomly determined crossover location. Moreover, a mutation factor is introduced to provide a certain probability of mutation for the underperforming group, thereby enhancing the algorithm’s ability to escape local optima. Comparative studies with traditional algorithms and other intelligent algorithms show that the AGPSO algorithm has a faster convergence speed, improves the stability of the integer ambiguity search results, and in practical experiments the baseline accuracy of the solution is within 0.02 m, which has some application value in the practical situation of short baselines.

## 2. GNSS Differential Positioning Model Analysis

### 2.1. Mathematical Model of Carrier Phase Double Difference

In high-precision positioning of GNSS, the term “single difference” refers to the disparity between carrier phase observations acquired from different stations while synchronously observing the same satellite. By employing a single difference, it becomes possible to eliminate satellite clock discrepancies, and, simultaneously, if the user and reference station are near equal altitudes, approximate zero values can be achieved for the ionospheric delay and tropospheric delay [40]. Consequently, this enables us to formulate the observation equation for carrier phase single difference [41]

(1) $$\phi_{ur}^{j}=\lambda^{-1}p_{ur}^{j}+f\delta_{ur}+N_{ur}^{j}+\varepsilon_{\phi ,ur}^{j}$$

where $\phi_{ur}^{j}$ denotes the carrier phase single difference observation between the two receivers *u*, *r* and the satellite *j* at the moment *t*; λ denotes the carrier wavelength; $p_{ur}^{j}$ denotes the geometric distance difference between the two receivers *u*, *r* and the satellite *j* at the moment *t*; f denotes the frequency; $\delta_{ur}$ denotes the clock difference between the two receivers *u*, *r* at the moment *t*; $N_{ur}^{j}$ denotes the differential integer at the moment *t*; and $\varepsilon_{\phi ,ur}^{j}$ denotes the other observation noise at the moment *t*.

The single-difference observation model can only partially mitigate the error parameter. Therefore, based on this model, an additional reference satellite is selected to calculate the observation difference between satellites, thereby obtaining the double difference observation equation. The double difference further eliminates the receiver clock difference in observations [42]. Consequently, for high-precision localization purposes, the carrier phase double difference model is commonly employed. The carrier phase double difference observation equation [43] can be expressed as follows:

(2) $$\phi_{ur}^{jk}=\lambda^{-1}p_{ur}^{jk}+N_{ur}^{jk}+\varepsilon_{\phi ,ur}^{jk}$$

where $\phi_{ur}^{jk}$ denotes the carrier phase double difference observation between the two receivers *u*, *r* and the satellites *j*, *k* at moment *t*; λ denotes the carrier wavelength; $p_{ur}^{jk}$ denotes the geometric distance difference between the two receivers *u*, *r* and the satellites *j*, *k* at moment *t*; $N_{ur}^{jk}$ denotes the double difference integer at moment t; and $\varepsilon_{\phi ,ur}^{jk}$ denotes the other observation noise at moment *t*. In estimating the ambiguity floating-point solution, $p_{ur}^{jk}$ is computed from the satellite position and receiver position, which can be obtained from the navigation message computation, and the receiver position can be obtained from the observation file computation.

### 2.2. Least Squares Estimation of Ambiguity Float Solutions

It is known from the double difference observation model that a double difference carrier phase observation equation has three unknowns and each additional co-viewing satellite will have one more double difference integer ambiguity, whereas the least-squares estimation can be easily extended to the case of multi-parameter estimation by solving an optimization problem to estimate all the parameters. For carrier phase-based GNSS positioning models, the mathematical models can all be reduced to the following linearized model [44]:

(3) y=Aa+Bb+e

where *y* is the carrier phase observation; *A* and *B* are both design matrices; *a* and *b* are the integer ambiguity vector and the baseline vector between the two base stations, respectively; and e denotes the observation error vector. The determination of the unknown parameters *a* and *b* in Equation (3) can be solved by least squares parameter estimation [45], therefore, Equation (3) is essentially a least squares solution problem, which can be expressed as:

(4) $$min\left\{∥y-Aa-Bb{∥}_{Q_{yy}}^{2}\right\}$$

where $Q_{yy}$ denotes the carrier phase variance covariance matrix. The float solution and baseline vectors and their corresponding covariance matrices are obtained by weighted least squares solving of Equation (4):

(5) $$\left[\begin{array}{c} \hat{a}\\ \hat{b} \end{array}\right]={\left[\begin{array}{cc} A^{T}Q_{yy}^{-1}A & A^{T}Q_{yy}^{-1}B\\ B^{T}Q_{yy}^{-1}A & B^{T}Q_{yy}^{-1}B \end{array}\right]}^{-1}\left[\begin{array}{c} A^{T}Q_{yy}^{-1}y\\ B^{T}Q_{yy}^{-1}y \end{array}\right]$$

(6) $$\left[\begin{array}{cc} Q_{\hat{a}\hat{a}} & Q_{\hat{a}\hat{b}}\\ Q_{\hat{b}\hat{a}} & Q_{\hat{b}\hat{b}} \end{array}\right]={\left[\begin{array}{cc} A^{T}Q_{yy}^{-1}A & A^{T}Q_{yy}^{-1}B\\ B^{T}Q_{yy}^{-1}A & B^{T}Q_{yy}^{-1}B \end{array}\right]}^{-1}$$

where $\hat{a}$ is the float solution; $\hat{b}$ is the baseline vector, $Q_{\hat{a}\hat{a}}$ is the covariance matrix corresponding to $\hat{a}$, and $Q_{\hat{b}\hat{b}}$ is the covariance matrix corresponding to $\hat{b}$. The floating-point solution can be computed in a single calendar element, but within a single calendar element, the floating-point solution may be subject to a variety of errors, and if a more accurate floating-point solution is required, more calendar elements are needed. Due to the integer nature of the ambiguity, the stationary solution of the integer ambiguity can be obtained by solving the minimum of the quadratic function of the following equation, i.e.,

(7) $$J\left(a\right)=min∥\hat{a}-a{∥}_{Q\hat{a}\hat{a}}^{2}$$

The ambiguity fixation process in Equation (7) is the process of searching for the integer vector with the shortest distance $\hat{a}$ in the integer space with $Q_{\hat{a}\hat{a}}$ as the weight and $\hat{a}$ as the center.

The first thing to be determined in the process of searching for integer ambiguity is the search space. All correct solutions must be encompassed within this search space. In practice, the baseline is usually the parameter vector that needs to be solved. For real-time localization of short baselines, the use of baseline length as a constraint can effectively reduce the scope of the search space and thus improve the efficiency of the algorithm, when the exact value of the baseline vectors is obtained beforehand using precision measurements. Assuming a given baseline length denoted as l, the scope of search can be expressed as follows:

(8) −lλ≤aurjk≤lλ

where λ is the GPS carrier wavelength and takes the value of 0.19 m.

### 2.3. Ambiguity Decorrelation

The ambiguous floating-point solution and its covariance matrix obtained by least squares estimation have a high correlation, in which case the search space is a narrow ellipsoid, which leads to inefficient search. Therefore, an ambiguity decorrelation operation should be performed before the integer of ambiguity search.

Among the ambiguous decorrelation algorithms, the integer Gaussian transform [46], LLL algorithm [47], and inverse integer Cholesky algorithm [48] are commonly used. Li et al. [49] evaluated the above three ambiguity decorrelation algorithms and proposed that for less than 20-dimensional low-dimensional covariance matrix inverse integer, the Cholesky algorithm has a better decorrelation effect. Since the dimensions of the ambiguity covariance matrix in GPS dynamic positioning are generally around 5–10 dimensions, this paper adopts the inverse integer Cholesky algorithm for ambiguity decorrelation, and the basic idea of this algorithm is to use the invertible integer transformation to transform the floating-point solutions and their covariance matrices, and the transformation process can be expressed as follows:

(9) $$\hat{z}=Z^{T}\hat{a}$$

(10) $$Q_{\hat{z}\hat{z}}=Z^{T}Q_{\hat{a}\hat{a}}Z$$

where Z is the integer transformation matrix; $\hat{z}$ is the floating-point solution after decorrelation; and $Q_{\hat{z}\hat{z}}$ is the covariance matrix after decorrelation. An integer least squares search is expanded using the transformed $\hat{z}$ and $Q_{\hat{z}\hat{z}}$ to determine the optimal integer candidate vector $\overset{˘}{z}$, and then an integer inverse transformation is used to determine the optimal integer candidate $\overset{˘}{a}$ for the ambiguity float solution:

(11) $$\overset{˘}{a}=Z^{-T}\overset{˘}{z}$$

(12) $$Q_{\overset{˘}{a}\overset{˘}{a}}=Z^{-T}Q_{\overset{˘}{z}\overset{˘}{z}}Z^{-1}$$

The ambiguity search is essentially defined as the minimum value of the equation within the search space. To enhance both the efficiency and stability of this search method, we propose in this paper to employ a genetic particle swarm optimization algorithm for conducting ambiguity searches.

## 3. Genetic Particle Swarm Optimization Algorithm Ambiguity Search

### 3.1. Classical Particle Swarm Optimization Algorithm

The particle swarm optimization (PSO) algorithm was introduced by Kennedy and Eberhart in 1995 AD, inspired by the study of birds’ flocking behavior. Its fundamental concept is to achieve optimal solutions through collaboration and information sharing among individuals within a group [50]. In the conventional particle swarm optimization algorithm, particles’ rate and position change equations are utilized.

(13) $$v_{id}^{k+1}=\omega v_{id}^{k}+c_{1}r_{1}\left(pbest-x_{id}^{k}\right)+c_{2}r_{2}\left(gbest-x_{id}^{k}\right)$$

(14) $$x_{id}^{k+1}=x_{id}^{k}+v_{id}^{k+1}$$

where 1≤i≤n; 1≤d≤n; k is the kth iteration; $c_{1}$ and $c_{2}\;$are the self-perception factor and the social-perception factor; $r_{1}$ and $r_{2}$ are random numbers on the interval [0, 1]; and ω is the inertia weight; pbest is the individual best; gbest is the global best.

The inertia weights ω play a crucial role in optimizing the performance of the PSO algorithm by effectively balancing the global exploration and local exploitation capabilities of the swarm. To enhance the global exploration capability during the early evolutionary stages, it is expected that larger values of particle velocities will be employed. Conversely, as the PSO algorithm progresses towards its later stages, where a strong local detection capability is required, smaller velocity values are anticipated. To address the issue of falling into local optima commonly encountered in standard particle swarm optimization algorithms, this study incorporates Hu’s [51] proposed method for decreasing inertia weight, thereby improving its effectiveness. The proposed approach aims to strike an optimal balance between global exploration and local exploitation through carefully selected inertia weights.

(15) $$\omega \left(k\right)=\omega_{max}-\left(\omega_{max}-\omega_{min}\right)\left(k/k_{max}\right)$$

where k is the current number of generations; $k_{max}$ is the maximum number of iterations.

### 3.2. Classical Genetic Algorithm

The genetic algorithm (GA), initially proposed by John Holland in the 1970s, is a computational model that simulates the process of biological evolution, incorporating natural selection and genetic mechanisms from Darwin’s theory. By employing mathematical operations and computer simulations, it transforms the problem-solving process into a series of chromosome gene crossovers, mutations, and other evolutionary processes. Essentially, GA is an efficient parallel global search method that autonomously acquires and accumulates knowledge about the search space while adaptively controlling the search process to identify optimal solutions.

The genetic algorithms encompass three fundamental operators: selection, crossover, and mutation. Selection functions as a mechanism for “choosing the best from the worst”. Crossover, in conjunction with selection rules, facilitates the preservation of valuable information while discarding unfavorable information. Mutation can produce new varieties that are indeed substantially different [52]. It is this distinctive search methodology that enables genetic algorithms to naturally evade the common pitfall of local minima encountered by other optimization algorithms.

### 3.3. Genetic Particle Swarm Optimization Algorithm

#### 3.3.1. Genetic Selection Cross-Mutation Operation

Due to the direct utilization of the PSO algorithm for resolving the integer ambiguity, it often suffers from premature convergence and gets trapped in local optimal solutions, significantly compromising the accuracy of the integer ambiguity solution [53]. In contrast, GA algorithms typically necessitate a substantial number of iterations and computations to discover an optimal solution, with potentially slow convergence particularly when dealing with large or complex problem spaces [54].

To address the aforementioned issues, this paper proposes an adaptive genetic particle swarm optimization (AGPSO) algorithm for single-frequency GNSS integer ambiguity-solving. The algorithm incorporates selection, crossover, and mutation operations from the genetic algorithm into the PSO algorithm, with specific enhancements as follows:

(1) The selection operation is responsible for identifying the dominant individuals within the current population. In this study, we adopt a meritocratic approach combined with half selection to identify the superior individuals in the population. Firstly, we calculate and rank their fitness levels, where higher fitness corresponds to higher ranking and increased probability of selection;
(2) Crossover operations are responsible for generating novel individuals, achieved by exchanging segments of their chromosomes to produce two offspring chromosomes. The position of crossover is determined randomly, increasing the likelihood of escaping local optima. In this study, the crossover was randomly performed on two selected individuals at two specific crossover points to create new individuals by combining information from the parent’s mating population;
(3) The mutation operation is responsible for facilitating the algorithm to escape from local optima, and a smaller value is generally chosen as the mutation probability. A higher mutation probability may lead to the destruction of optimal solutions. After conducting numerous experiments, a mutation probability of 0.1 was adopted in this paper.

#### 3.3.2. AGPSO Adaptation Function Establishment

The fitness function of AGPSO utilizes the principle of least squares to fit a fixed solution with an ambiguous floating-point solution after reduced correlation so that the floating-point solution reaches the integer optimum under the least squares criterion. The AGPSO population is optimized according to the size of the fitness. The objective function of the integer least squares estimation of integer ambiguity can be obtained according to Equation (7):

(16) $$J\left(N\right)={\left(\hat{N}-N\right)}^{T}Q_{\hat{N}}^{-1}\left(\hat{N}-N\right)$$

where $\hat{N}$ is the ambiguity float solution; N is the integer ambiguity vector. $Q_{\hat{N}}^{-1}$ is the inverse matrix of the covariance matrix corresponding to $\hat{N}$. Teunissen [55] gave a probabilistic proof using integer least squares estimators. Introducing the admissible integer estimator and generalizing the classical leveling theory proves that the integer LS estimator is the best in the sense of maximizing the probability of correct integer estimation, but most of the value domains of the objective function (16) are relatively flat, with very little change in the function values, and even if *N* undergoes a small change, the function values are still relatively stable, which makes the search inefficient, so the objective function (16) is improved by proposing the following fitness function:

(17) $$F\left(N\right)=b-\lambda_{1}log\left(J\left(N\right)\right)-\lambda_{2}{||\hat{N}-N||}^{2}$$

where b is a large constant; $\lambda_{1},\;\;\lambda_{2}$ are the weights of the performance indicators. log function is more sensitive to the change of J(N), while log function has the nature of smoothing, which helps to reduce the problem of local extremes in the search space, and it is easier to find the global optimal solution. When J(N) is smaller, the value of F(N) is larger, i.e., the accuracy of the solution is higher. Therefore, by maximizing the objective function, the estimation of the integer’s ambiguity can be made more accurate. The ${||\hat{N}-N||}^{2}$ emphasizes the size of the residual term, which penalizes the fitting error of the integer ambiguity vector. When the residual term is large, the fitness function decreases, thus encouraging the search algorithm to better fit the known integer ambiguity vector N. In the simulation, Equation b is 100, and $\lambda_{1}$ and $\lambda_{2}$ are 0.7 and 0.3, respectively.

#### 3.3.3. Basic Flow of AGPSO

**Figure 1:**
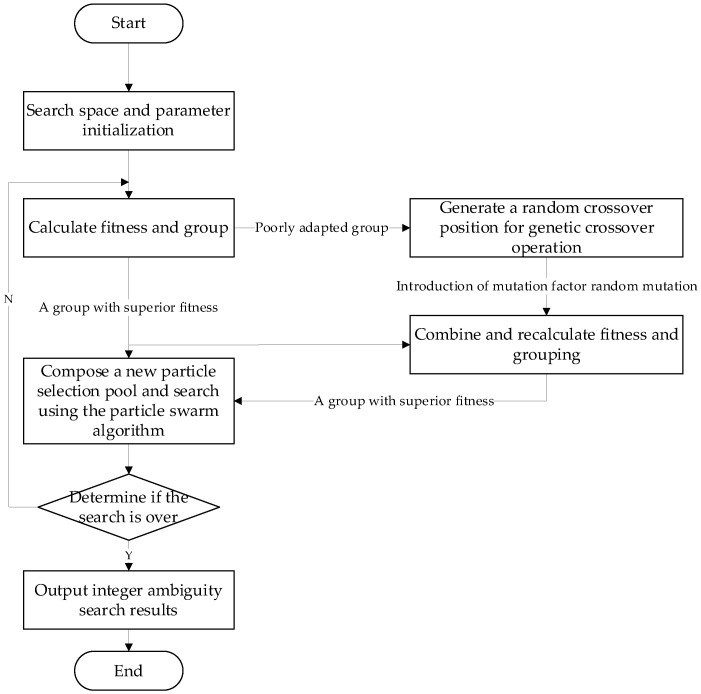
Flowchart of AGPSO algorithm.

The flowchart of the AGPSO algorithm is shown in Figure 1 and described as follows:

(1) Initialize the velocity and position of the particles, the maximum and minimum values of the weights, the acceleration constant, the population size, the Mutation factor, the maximum number of iterations, and the minimum error for the termination of the algorithm;
(2) The initial adaptation value of each particle is calculated, the population is divided into two groups with good and poor adaptation, and the group with good adaptation is selected to enter the next generation;
(3) A random crossover position is generated, and a crossover operation is performed on the poorly adapted set;
(4) Introducing a mutation factor that randomly mutates the poorly adapted group when the random number is smaller than the mutation factor;
(5) After the group with poor fitness undergoes the cross-mutation operation, the particle fitness is recalculated, combined with the group with good fitness initially, and reclassified into two groups of good and poor fitness. The re-grouped group with good fitness and the initial group with good fitness are taken to form a new particle selection pool;
(6) Use the best-adapted value in the new particle pool as the global best optimum, and use the position corresponding to this adapted value as the global optimum position of the particle;
(7) Update the particle velocity by the previous formula and limit the flight speed so that it cannot exceed the maximum flight speed;
(8) Update the particle position by the previous formula and compare whether the adapted value of each particle is better than the historical optimal value; if yes, then replace it;
(9) Calculate whether the adapted value of the particle’s global optimum is better than the historical optimum, and if so, replace it;
(10) Repeat 2–9 until the set minimum error is met or the maximum number of iterations is reached;
(11) Output the global optimal value of the optimal particle and its corresponding position as well as the local optimal value and corresponding position of each particle.

## 4. Numerical and Experimental Analysis

### 4.1. Numerical Analysis

To assess the feasibility and performance of the adaptive genetic particle swarm optimization (AGPSO) algorithm proposed in this study for solving ambiguity problems, a comparative analysis was conducted between classical algorithms, particle swarm optimization algorithms, and genetic particle swarm optimization algorithms.

**Figure 2:**
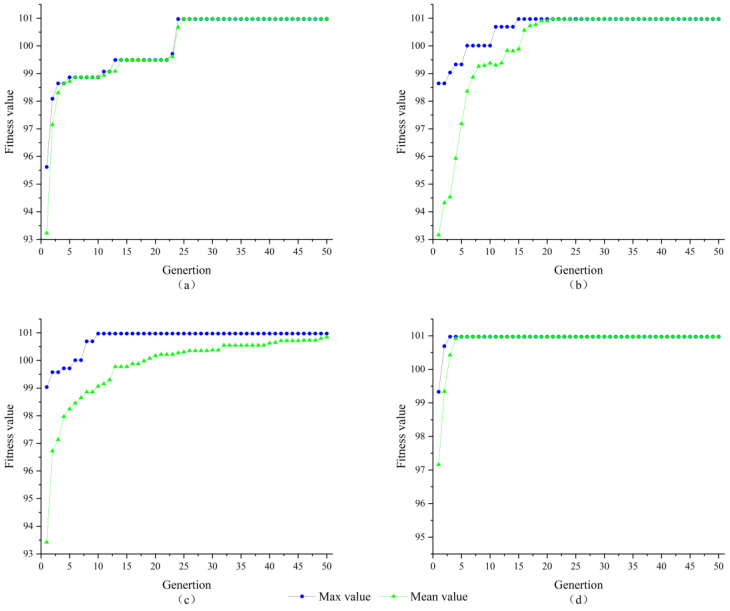
Three-dimensional integer ambiguity solving results of (**a**) SA algorithm; (**b**) GA algorithm; (**c**) PSO algorithm; (**d**) AGPSO algorithm.

In this study, three classical intelligent algorithms, namely Simulated Annealing Algorithm (SA), Genetic Algorithm (GA), and Particle Swarm Optimization Algorithm (PSO), were selected as comparison algorithms. The reference index used to evaluate their performance is the ability to converge to the optimal solution quickly within a specified number of iterations. To ensure consistent experimental conditions, we set the dimension D of integer ambiguity to 3, overall size k to 20, iteration termination at 50, $c_{1}$ and $c_{2}$ at 2.05, and adjusted inertia weights ω in the range of 0.4 to 0.9. Equation (17) represents the fitness function used in this study. To simulate real-world scenarios, we adopted a well-known arithmetic example proposed by Jonge [56]. Figure 2 presents the results of four algorithms for solving three-dimensional integer ambiguity.

**Figure 3:**
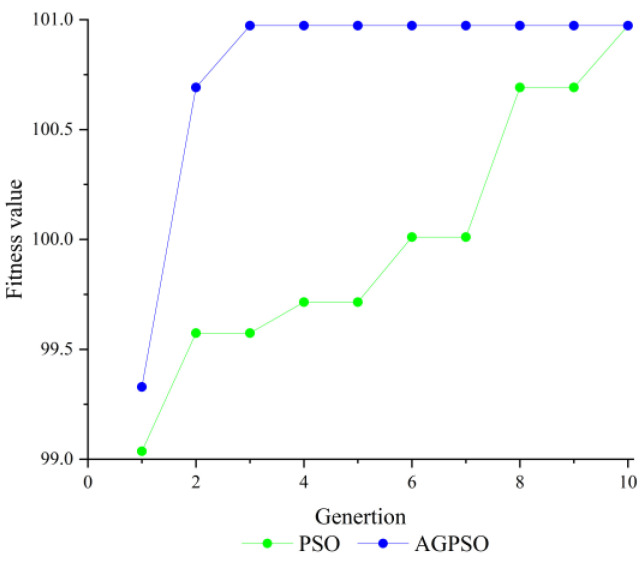
Results of the first ten generations of the 3D integer ambiguity solving.

From the comparison results in Figure 2, it can be seen that SA has the slowest convergence speed, and the SA algorithm converged to the optimal solution after 24 iterations; the GA algorithm is slightly faster than the SA algorithm and converged to the optimal solution after the 15th iteration, the PSO algorithm reached the optimal solution in the 11th iteration compared to the GA algorithm, and the AGPSO algorithm converged to the optimal solution in the 3rd iteration, and it is the best performer among the four algorithms. Therefore, the integer ambiguity solution, shows that the AGPSO algorithm has better search effectiveness compared to other classical algorithms. Figure 3 exemplifies the evolution of the best individual for the first ten iterations in a single solution for both PSO and AGPSO algorithms.

**Figure 4:**
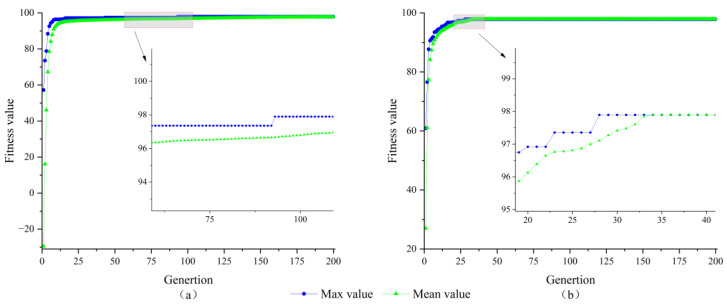
Twelve-dimensional integer ambiguity solving results (**a**) PSO algorithm; (**b**) AGPSO algorithm.

From Figure 3, it can be seen that the convergence speed of the PSO algorithm is much lower than that of the AGPSO algorithm and it is easy to fall into the local optimum, which shows that the AGPSO algorithm is more likely to jump out of the local optimum than PSO. To further explore the performance of the PSO algorithm and AGPSO algorithm, twelve-dimensional algorithms are used for simulation. The parameters of the algorithms are shown in Table 1, and the twelve-dimensional integer ambiguity-solving results of the two algorithms are shown in Figure 4.

**Figure 5:**
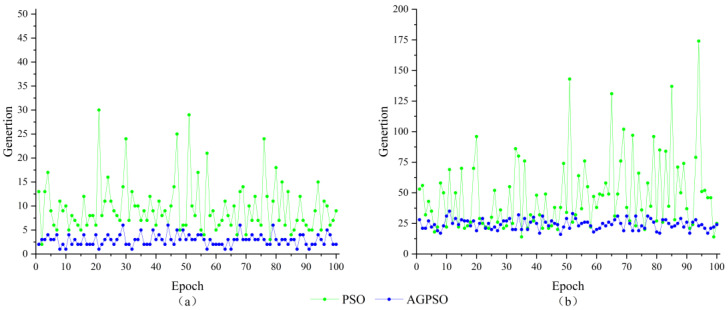
100 times integer ambiguity solving results; (**a**) three-dimensional; (**b**) twelve-dimensional.

As can be seen from the curves in Figure 4, both algorithms converge to the global optimum, the PSO algorithm is trapped in the local optimum until it reaches the optimal solution in the 93rd iteration, while the AGPSO algorithm converges to the optimal solution in the 28th iteration. It shows that the AGPSO algorithm still has better search effectiveness in the high-dimensional case. To eliminate the eventuality of the test and prove the AGPSO algorithm’s search reliability, 100 consecutive searches for three-dimensional and 12-dimensional integer ambiguity were performed using the PSO algorithm and the AGPSO algorithm. The number of iterations required for both algorithms to converge to the optimal solution is recorded in Figure 5.

It can be seen from Figure 5 that the number of iterations required for the PSO algorithm to converge to the optimal solution in three-dimensional integer ambiguity solving is about 10 iterations, while the average number of iterations required by the AGPSO algorithm is about 5 iterations. In twelve-dimensional integer ambiguity, solving a PSO algorithm requires about 50 iterations to reach the optimal solution, while the average number of iterations required by the AGPSO algorithm is about 25. In terms of convergence speed, the AGPSO algorithm is about twice as fast as the PSO algorithm, which verifies the reliability of the AGPSO algorithm. The AGPSO algorithm solves the problem that the PSO algorithm is easy to fall into local optimization.

From the principle of the algorithm, the adaptive weights of AGPSO gradually decrease during the iteration process, which reduces the search range of the algorithm when the individual population tends to mature. In addition, the optimal half-taking strategy, the stochastic crossover strategy, and the stochastic mutation strategy all enable the algorithm to jump out of the local optimum quickly.

### 4.2. Test Analysis

**Figure 6:**
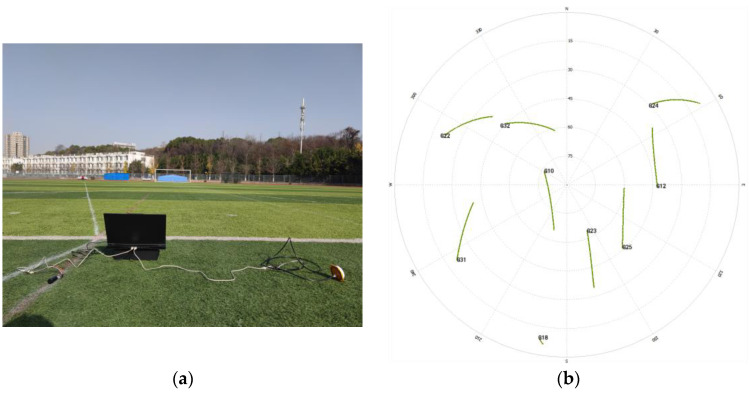
1.42m baseline test environment. (**a**) Test scenario; (**b**) satellite zenith distribution map.

**Figure 7:**
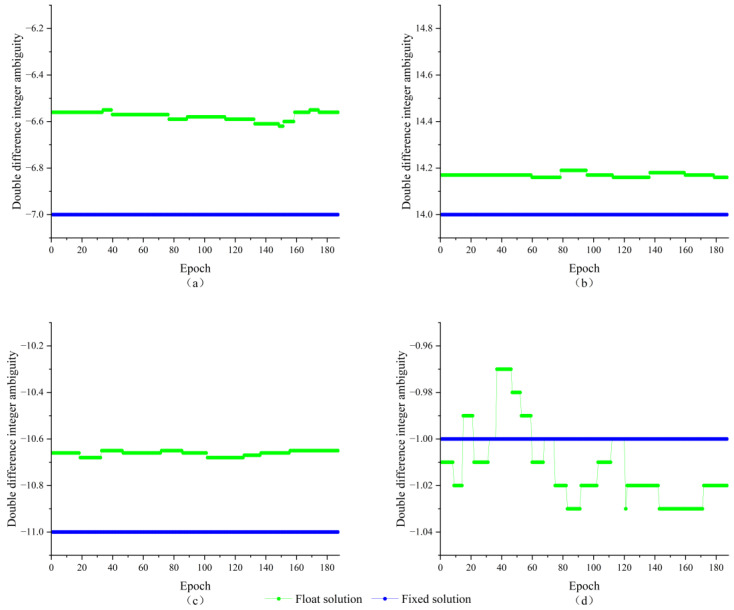
Results of double difference ambiguity solving; (**a**) Satellite 12-10; (**b**) Satellite 23-10; (**c**) Satellite 25-10; (**d**) Satellite 32-10.

To verify the applicability and effectiveness of the algorithm in practical situations. In the new playground of Nanjing Forestry University, two satellite receivers are used to form a short baseline of 1.42 m. The test scene is shown in Figure 6a, with a sampling interval of 5 s for the receivers, a satellite cutoff altitude angle of 30°, and a continuous observation of about 20 min, obtaining 187 valid calendar elements. Firstly, a single-point localization is performed for the two antennas, which is processed by double difference; the floating-point solution and its covariance matrix are obtained with the help of weighted least squares estimation, and finally the AGPSO algorithm is used to search for the integer ambiguity after double difference. The whole process of double difference integer ambiguity solving is usually carried out in calendar element by calendar element. Each calendar element is processed independently to find the appropriate integer ambiguity solution, and then the process is continued in subsequent calendar elements, gradually converging to the exact value over multiple iterations of calendar elements. For GPS L1 single-frequency signals, the satellite zenith distribution map during the acquisition period is shown in Figure 6b, with five effective observation satellites, and the reference satellite is selected to be the No. 10 satellite with the highest altitude angle during the observation period, which is composed of double difference ambiguity with the satellites of No. 12, No. 23, No. 25, and No. 32, and the result of the integer ambiguity solving is shown in Figure 7.

**Figure 8:**
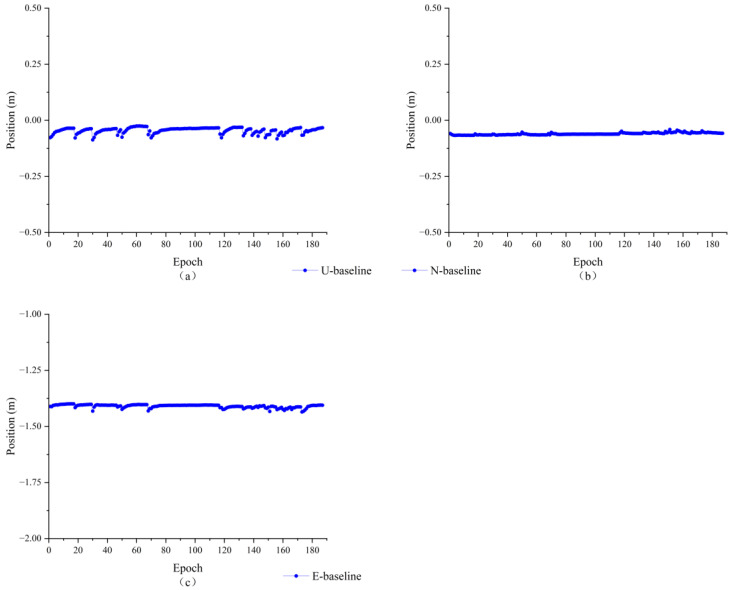
1.42m baseline solution results (**a**) upward direction (**b**) northward direction (**c**) eastward direction.

As shown in Figure 7, the double difference integer ambiguities are composed of satellite 12, 23, 25, and 32 and satellite 10 are −7, 14, −11, and −1, respectively, and all of them have completed the ambiguity fix. Since the length of the baseline is known and the wavelength of the GPS L1 carrier is also known to be about 0.1903 m, the baseline solution is used to verify the results of the double difference perimeter ambiguity solution. The baseline solution results are shown in Figure 8.

**Figure 9:**
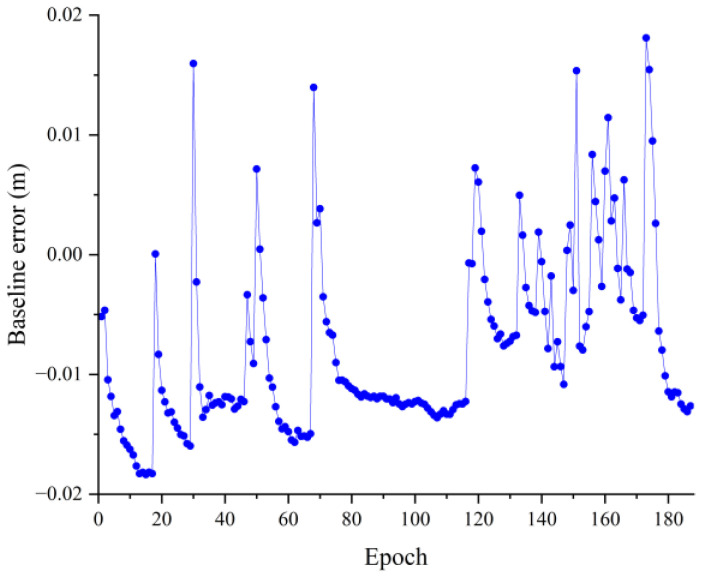
1.42 m baseline solution error.

As can be seen in Figure 8, the relative positions in the three directions are largely stable. However, the position components in the U and E directions are not smooth and are split into many parts by hops. It is possible that the antenna’s close proximity to the ground caused a large multipath effect, resulting in the generation of multiple hops. Figure 9 shows the baseline solving error, from which it can be seen that the baseline solving error is within 0.02 m. Therefore, it can be recognized that the double difference integer ambiguity search is correct, indicating that the AGPSO algorithm also has applicability and validity in practical situations, but there were still more jumps.

**Figure 10:**
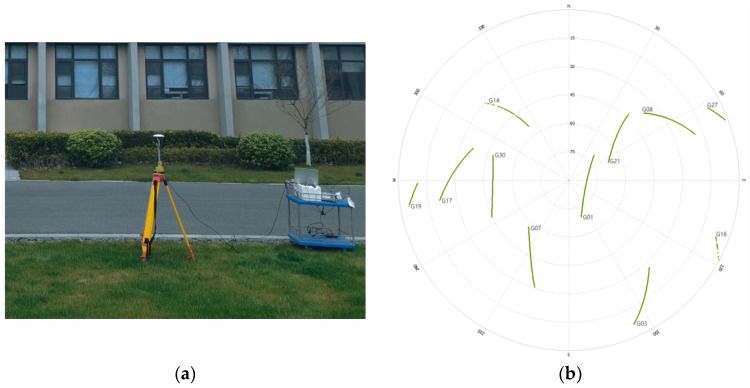
263.52m baseline test environment. (**a**) Test scenario; (**b**) satellite zenith distribution map.

**Figure 11:**
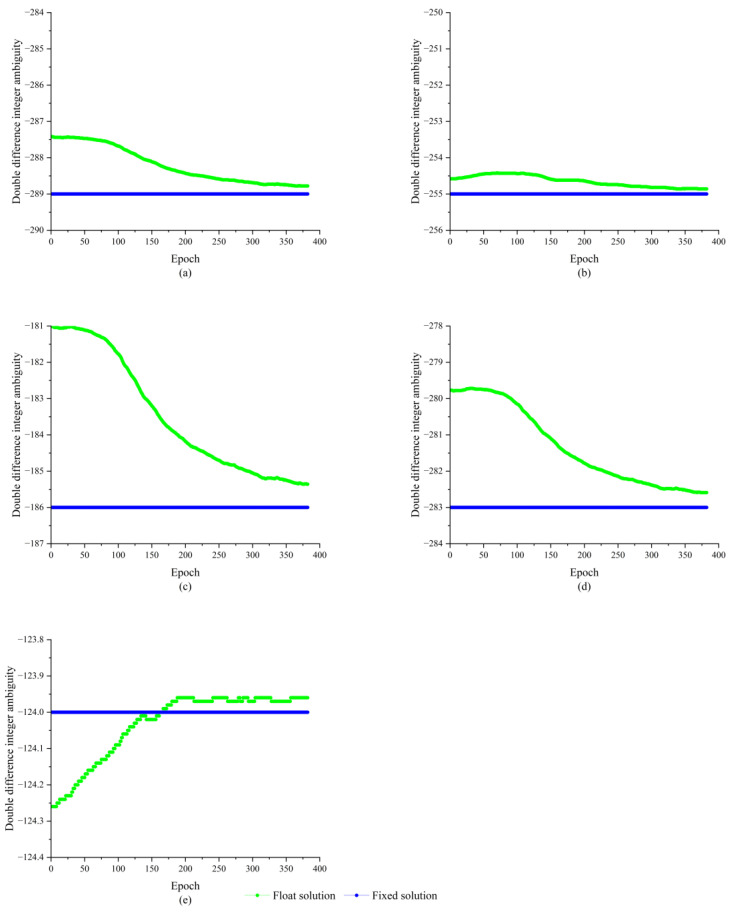
Results of double difference ambiguity solving; (**a**) Satellite 7-1; (**b**) Satellite 8-1; (**c**) Satellite 17-1; (**d**) Satellite 30-1; (**e**) Satellite 21-1.

In response to these problems, the test scenario was reconstructed, while the baseline length was increased to 263.52 m. The test scenario is shown in Figure 10a, and for the GPS L1 single-frequency signal, the satellite zenith distribution map during the acquisition is shown in Figure 10b. The observation time lasts 40 min, the data sampling rate is 5 s, the satellite cutoff altitude angle is 15°, and 382 effective calendar elements are acquired. For the GPS L1 single-frequency signal, there are six effective observation satellites, and the reference satellite is selected to be the No. 1 satellite with the highest altitude angle during the observation period, which is composed of double difference ambiguity with the satellites of No. 7, No. 8, No. 17, No. 30, and No. 21, and the results of ambiguities solving for the integer are shown in Figure 11.

**Figure 12:**
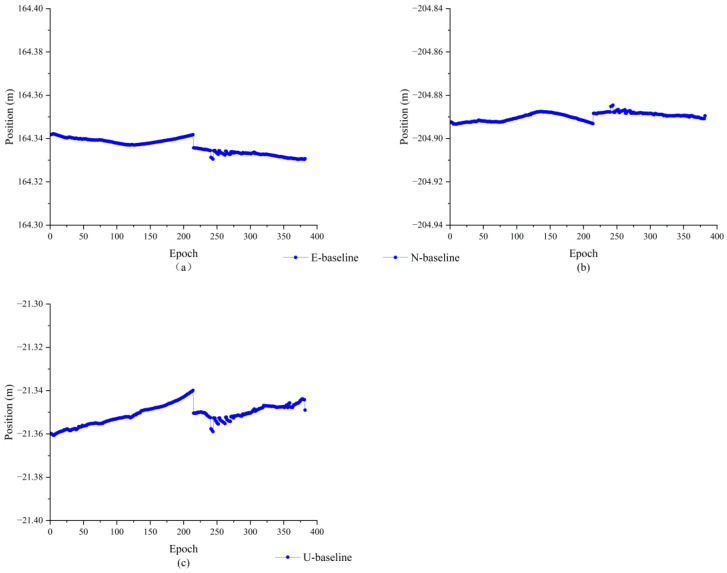
263.52 m baseline solution results; (**a**) upward direction; (**b**) northward direction; (**c**) eastward direction.

As shown in Figure 11, the double difference integer ambiguities composed of satellite 7, 8, 17, 30, and 21 and satellite 1 are −289, −255, −186, −283, and −124, respectively, and all of them have completed the ambiguity fixation. Since the length of the baseline is known and the wavelength of the GPS L1 carrier is also known to be about 0.1903 m, the baseline solution is used to verify the results of the double difference integer ambiguity solution. The baseline solution results are shown in Figure 12.

**Figure 13:**
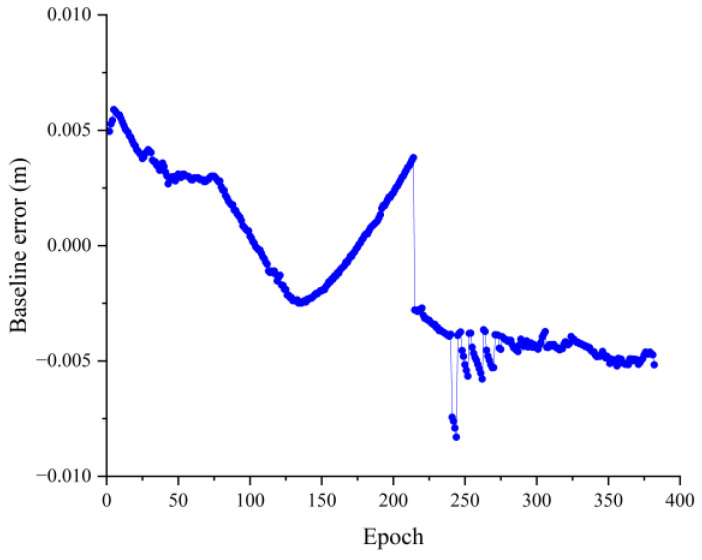
263.52 m baseline solution error.

As can be seen from Figure 12, the relative positions in all three directions are more stable, and the number of jumps has been significantly reduced. For the phenomenon of individual jumps that still exists, subsequent attempts can be made to reduce the jumps by improving the quality of receivers, increasing the number of satellites, and reducing the obstacle blockage. Figure 13 shows the baseline solving error, from which it can be seen that the baseline solving error is within 0.01 m. Therefore, it can be concluded that the double difference integer ambiguity search is correct, indicating that the AGPSO algorithm has applicability and validity in practical situations as well.

If the fixed integer ambiguity vector is inaccurate, not only will it not improve the localization accuracy, but also reduce the localization accuracy. Therefore, when the fixed integer ambiguities vector is fixed, it also needs to be examined, i.e., the ambiguities are confirmed. On the basis of systematic study of the theory of ambiguities solution, Verhagen et al. [5] proposed a method based on the fixed failure rate that is often used in conjunction with the traditional differentiation test method, but the threshold value of the differentiation test method based on the fixed failure rate method also needs to be calculated for a large number of samples statistically, which restricts the practical application of this method. The Ratio value is one of the most commonly used methods to test the reliability of integer ambiguities fixation, which is obtained by the ratio of the sum of squares of the residuals of the suboptimal ambiguities vector to the sum of squares of the residuals of the optimal ambiguities vector [57], and according to the experience, the ratio value is standing for 3. When the ratio value is greater than 3, it is determined that the ambiguities fixation solution is correct. Then, the success rate = number of fixed correct calendar elements/total number of calendar elements. To further validate the reliability of the algorithms, the success rates of solving the double difference ambiguity using the LAMBDA algorithm and AGPSO algorithm were counted separately. Since the results of the test with a baseline length of 1.42 m produced multiple jumps and the baseline length is too short, it cannot reflect the function of the algorithm, and its success rate cannot be used as a reference. Only the success rate of ambiguity resolution for the measured data with a baseline length of 263.52 m was counted, and the resolution results are shown in Table 2.

The results in Table 2 show that the success rate of solving using the LAMBDA algorithm is 96.15%, the success rate of solving using the proposed AGPSO algorithm is 95.88%, and there is not much difference between the success rates of the two algorithms. Therefore, the AGPSO algorithm also has high reliability and practicability under short baseline practical applications.

## 5. Conclusions

In this paper, the important problem of integer ambiguity solving is investigated. Aiming at the existing ambiguity solving algorithms, which still have shortcomings such as low convergence speed and instability, the AGPSO algorithm is proposed to find the optimal solution of the integer ambiguity, which introduces the optimal half-taking, random crossover, and mutation operations to make the algorithm jump out of the local optimal solution more easily, and the improved fitness function to improve the convergence and search performance of the algorithm, and to improve the stability of the ambiguity solving results. Finally, experiments have been carried out using simulation data and measured data, as can be seen from the experimental results:

(1) In the integer ambiguity search, the particle swarm optimization algorithm (PSO) converges faster than the SA and GA algorithms, and the AGPSO algorithm can reach the optimal solution faster compared with the PSO algorithm. Through high-dimensional data simulation, it is verified that the proposed AGPSO algorithm can effectively solve the problem that the PSO algorithm is easy to fall into the local optimum and improves the efficiency of the integer ambiguity search;
(2) To eliminate the chance of the test, the PSO algorithm and the AGPSO algorithm were used to search the three-dimensional and twelve-dimensional integer ambiguity 100 times consecutively. The results show that the AGPSO algorithm approximately doubles the convergence speed of the PSO algorithm, and the AGPSO algorithm jumps out of the local optimum more easily than the PSO algorithm, which significantly improves the stability of the results of the integer ambiguity solution;
(3) For GPS L1 single-frequency signal, the AGPSO algorithm is used to search the integer ambiguity after double difference and carry out the baseline solving, the solving result shows that the baseline error is within 0.02 m, which verifies the applicability and validity of the AGPSO algorithm in the practical application. The AGPSO algorithm can be a very good solution to the short baseline solving of the integer ambiguity of searching the problem of inefficiency and instability.

## Figures and Tables

**Table 1:** Parameter settings of the two algorithms.

Parameters	Value
$\mathrm{Learning}\;\mathrm{factor}\;\;c_{1}$	2.05
$\mathrm{Learning}\;\mathrm{factor}\;\;c_{2}$	2.05
Inertia coefficient ω	0.4–0.9
Population size m	100
Particle dimension D	12
Number of iterations k	200

**Table 2:** Success rates of the two algorithms solvers.

Base Line (m)	Method	Epochs	Success Epochs	Success Rate (%)
263.52	LAMBDA	364	350	96.15
	AGPSO	364	349	95.88

## Data Availability

Data are contained within the article.
